# Seasonal groundwater quality assessment and irrigation suitability in coastal aquifers of Puri District, Odisha, India

**DOI:** 10.1038/s41598-026-39145-8

**Published:** 2026-02-09

**Authors:** Yugajyoti Barik, Abinash Dalai, Sheelabhadra Mohanty, Vinod Kumar Tripathi, Sasmita Chand

**Affiliations:** 1 ICAR-Indian Institute of Water Management, Bhubaneswar, Odisha India; 2 Institute of Agricultural Science, Banaras Hindu University, Varanasi, Uttar Pradesh India; 3 Manipal School of Architecture and Planning, Manipal Academy of Higher Education, Manipal, Karnataka India

**Keywords:** Groundwater, Kelly’s ratio, Sodium adsorption ratio (SAR), Water quality index (WQI), WHO, Environmental sciences, Hydrology

## Abstract

Assessing seasonal groundwater quality in coastal aquifers is critical for ensuring sustainable drinking water supply and irrigation management under increasing anthropogenic and climate-induced pressures. This study evaluates the spatio-temporal variation of groundwater quality across four hydrological seasons winter (December-February), pre-monsoon (March-May), monsoon (June–September), and post-monsoon (October-November) in the coastal aquifers of Puri district, Odisha, India, during 2021–2022. Groundwater samples collected from twelve representative monitoring locations were analyzed for major physicochemical parameters, including pH, EC, TDS, total hardness, major cations (Ca²⁺, Mg²⁺, Na⁺, K⁺), and major anions (Cl⁻, SO₄²⁻, HCO₃⁻, NO₃⁻, and F⁻). Drinking water suitability was assessed using BIS (2012) and updated WHO (2022) guidelines, while irrigation suitability was evaluated using Sodium Adsorption Ratio (SAR), Kelly’s Ratio, EC, TDS, and USSL classification. The Water Quality Index (WQI) was employed to integrate multiple parameters into a single indicator of potability. Results reveal that groundwater quality ranges from good to excellent across most seasons, with localized deterioration during pre-monsoon and monsoon periods due to salinity enrichment and anthropogenic inputs. Irrigation indices indicate predominantly low to moderate sodium and salinity hazards, confirming suitability for agricultural use. The study provides a comprehensive seasonal and multi-index assessment of groundwater quality in a sensitive coastal aquifer system and contributes to SDG-6 (Clean Water and Sanitation) and SDG-13 (Climate Action) by supporting evidence-based groundwater management and climate-resilient water planning.

**Supplementary Information:**

The online version contains supplementary material available at 10.1038/s41598-026-39145-8.

## Introduction

Water is a critical and irreplaceable natural resource that sustains all forms of life and supports essential human activities such as agriculture, industry, and domestic use^1,2^. Beyond meeting direct human demands, groundwater plays a vital ecological role by sustaining base flow in rivers, lakes, and wetlands, thereby maintaining aquatic ecosystems and water quality during dry periods^3,4^. Despite its importance, groundwater resources worldwide are under increasing stress due to over-extraction, unregulated land use, and contamination from natural and anthropogenic sources. These pressures threaten groundwater sustainability and highlight the urgent need for effective monitoring, management, and conservation strategies^5,6^.

Groundwater is widely recognized as the most reliable source of freshwater for drinking and irrigation, particularly in regions where surface water availability is seasonal, limited, or unpredictable. This dependence is especially pronounced in coastal regions, where groundwater often serves as the primary freshwater source. However, coastal aquifers are inherently vulnerable to quality deterioration due to their proximity to seawater and their sensitivity to both natural processes and human activities^7^. Seawater intrusion, excessive groundwater abstraction, agricultural intensification, rapid urbanization, and climate-driven hydrological variability collectively pose serious risks to groundwater quality in coastal environments. Recent studies emphasize that seasonal recharge processes play a critical role in controlling groundwater chemistry in such systems, making multi-seasonal monitoring indispensable for understanding hydrogeochemical behavior and ensuring sustainable resource management^8,9^.

Water is fundamental to life, and even the most basic cellular processes and physiological functions depend on its availability and quality^10,11^. As the global population continues to grow, the demand for water has increased sharply to support expanding food production, industrial development, and urbanization. Among all available freshwater sources, groundwater stands out as the most significant and dependable source for human consumption, particularly in areas where surface water is either scarce or highly variable. Globally, groundwater supplies drinking water to billions of people and underpins agricultural irrigation systems that are essential for food security. Additionally, groundwater contributes to ecosystem stability by maintaining hydrological connectivity between surface and subsurface water bodies. However, the growing imbalance between groundwater withdrawal and natural recharge, combined with contamination from agricultural runoff, industrial effluents, and domestic waste, has raised serious concerns regarding the long-term availability and integrity of groundwater resources^5,6^.

India presents a compelling case of groundwater dependence and vulnerability. With nearly 18% of the global population, India is the largest consumer of groundwater in the world, extracting approximately 230 cubic kilometers annually over one-quarter of the global total (World Bank 2012). Recent assessments estimate India’s total annual groundwater recharge at 437.60 billion cubic meters (BCM), with extractable resources of 398.08 BCM and actual extraction of 293.16 BCM^12^. Despite occupying only 2.4% of the world’s land area, India utilizes around 4% of the global freshwater supply. This extensive reliance on groundwater for agricultural, industrial, and domestic purposes underscores the need for comprehensive and scientifically robust groundwater quality monitoring and management frameworks to mitigate risks associated with over-exploitation and contamination^13,14^.

Within India, coastal states face particularly acute groundwater challenges due to salinization, climate variability, and intense human activity. In the eastern coastal state of Odisha, the Puri district represents a hydrogeologically sensitive coastal alluvial system influenced by the Bay of Bengal. The district’s unique topography, climatic conditions, and land-use patterns create multiple stressors on groundwater quality. Factors such as heavy tourist inflow, agricultural runoff, industrial discharges, and saltwater intrusion contribute significantly to groundwater quality deterioration in this region^15,16^. Moreover, seasonal variations in rainfall, evapotranspiration, and recharge exert strong controls on groundwater chemistry, resulting in marked temporal fluctuations. Understanding these seasonal dynamics is essential for accurately assessing groundwater quality and identifying periods of heightened vulnerability in coastal aquifers^17,18^.

In the coastal aquifer system of Puri district, groundwater quality is governed by complex interactions between geological formations, seawater intrusion, anthropogenic activities, and monsoonal recharge processes. To capture these dynamics, twelve representative groundwater sampling locations were selected across the coastal region, and samples were collected during four distinct hydrological seasons: winter, pre-monsoon, monsoon, and post-monsoon. The study evaluates key physicochemical parameters, including pH, electrical conductivity (EC), total dissolved solids (TDS), total hardness (TH), calcium, magnesium, sodium, potassium, nitrate, carbonate, fluoride, and bicarbonate. Groundwater quality was assessed for drinking purposes by comparing observed concentrations with Bureau of Indian Standards (BIS 2012) and World Health Organization (WHO 2021, updated to WHO 2022) guidelines. Irrigation suitability was evaluated using widely accepted indices such as Kelly’s Ratio, Sodium Adsorption Ratio (SAR), EC, and TH. Additionally, the Water Quality Index (WQI) was employed to integrate multiple parameters into a single indicator, providing an overall assessment of groundwater potability. The results indicate that groundwater quality in the study area is generally potable and ranges from good to excellent across all four seasons, while also being largely suitable for irrigation.

Although groundwater quality in Odisha’s coastal regions has been the focus of several studies, there remains a notable gap in comprehensive seasonal assessments specifically addressing both drinking and irrigation suitability in the Puri district. Most existing studies are either limited to single-season analyses or focus on isolated parameters such as salinity, nitrate, or cation anion balance, with insufficient emphasis on spatiotemporal variability across all hydrological seasons. Furthermore, integrated evaluations combining multiple indicators such as WQI, SAR, Kelly’s Ratio, TDS-based USGS classification, hardness classification, palatability criteria, and GIS-based spatial mapping are scarce for this coastal aquifer system. Consequently, the combined influence of anthropogenic inputs, saline intrusion, and monsoonal recharge on groundwater suitability remains inadequately understood.

Therefore, a methodical and multi-index approach is essential to comprehensively understand groundwater dynamics, identify seasonal vulnerabilities, and support sustainable groundwater management in the coastal aquifers of Puri district. Accordingly, the aim of this study is to evaluate the seasonal groundwater quality and irrigation suitability of the coastal aquifers of Puri district using an integrated hydrochemical and index-based framework. The specific objectives are to: (i) analyze seasonal variations in major physicochemical parameters across four hydrological seasons; (ii) assess drinking water suitability using BIS (2012) and updated WHO (2022) standards; (iii) evaluate irrigation suitability using SAR, Kelly’s Ratio, EC, TDS, and USSL classification; and (iv) integrate Water Quality Index (WQI) and GIS-based spatial analysis to identify seasonal and spatial vulnerability zones.

## Materials and methods

Groundwater samples were collected from all selected locations during four distinct hydrogeological seasons Winter (Dec-Feb), Pre-monsoon (Mar-May), Monsoon (Jun-Sep), and Post-monsoon (Oct-Nov) to assess temporal variations in water quality and irrigation suitability. Standard field procedures were followed during sampling, preservation, and transportation of samples to the laboratory. Physicochemical parameters were analyzed using APHA (2017) protocols, and the results were compared with national and international drinking water guidelines provided by BIS (2014) and WHO (2021). The overall potability of groundwater was evaluated using the Water Quality Index (WQI), while irrigation suitability was determined through Sodium Adsorption Ratio (SAR), Kelley’s Ratio, and other statistical indices. Groundwater classification was further carried out based on the USGS system for Total Dissolved Solids (TDS) and the WHO (1996) palatability criteria, along with hardness-based categorization to determine the degree of mineralization. Descriptive and inferential statistical analyses were performed to interpret the seasonal variability in water chemistry. Spatial distribution maps for all major parameters and indices were generated for each season using GIS interpolation techniques to visualize spatial trends and identify zones of concern. This integrated methodological approach enabled a comprehensive assessment of groundwater quality dynamics across the study area. Field measurements of pH and EC were conducted using calibrated portable meters. Laboratory analyses followed APHA (2017) standard methods, including flame photometry for Na⁺ and K⁺, EDTA titration for Ca²⁺ and Mg²⁺, spectrophotometric methods for NO₃⁻ and SO₄²⁻, and ion-selective electrodes for fluoride. Spatial distribution maps were generated using ArcGIS with Inverse Distance Weighting (IDW) interpolation.

### Study area and sampling

Puri district, located in eastern Odisha, is a coastal alluvial region that is predominantly rural, with a population of approximately 1.70 million and a population density of 488 persons per km². The district forms part of the Mahanadi River basin and supports a net sown area of about 0.11 million hectares, producing nearly 0.43 million tonnes of paddy, pulses, oilseeds, and other agricultural crops. Agriculture is practiced across three seasons in command areas and two seasons in non-command areas, with paddy being the dominant crop. The irrigation potential of the district is estimated at 0.12 million hectares during the kharif season and 0.08 million hectares during the rabi season.

The drainage system of the district is also part of the Mahanadi River basin and exhibits pronounced seasonal fluctuations. The south-west monsoon serves as the primary source of precipitation for the region. The district receives an average annual rainfall of 1449.1 mm (NAQUIM Report 2018), of which nearly 75% occurs between June and September. Flooding is a frequent phenomenon in the district, largely due to the high dependency on the south-west monsoon, which is inherently unpredictable.

An analysis of 24 years of rainfall data spanning the period from 1982 to 2020 indicates substantial interannual variability, with the highest recorded rainfall of 2146 mm occurring in 1991 and the lowest rainfall of 522 mm observed in 1974. The region experiences a subtropical climate characterized by pleasant winters and intensely hot, dry summers. The warm season extends from March to mid-June, followed by the rainy season from June to September, while the winter season typically lasts from November to the end of February. Puri district experiences high relative humidity throughout much of the year, with wind speeds increasing during the summer and south-west monsoon periods. Owing to its coastal location, the district benefits from cooler sea breezes, which moderate the otherwise tropical summer temperatures. Summers generally last from March to June, winters from October to January, and moderate temperatures prevail during July and August.

**Fig. 1:**
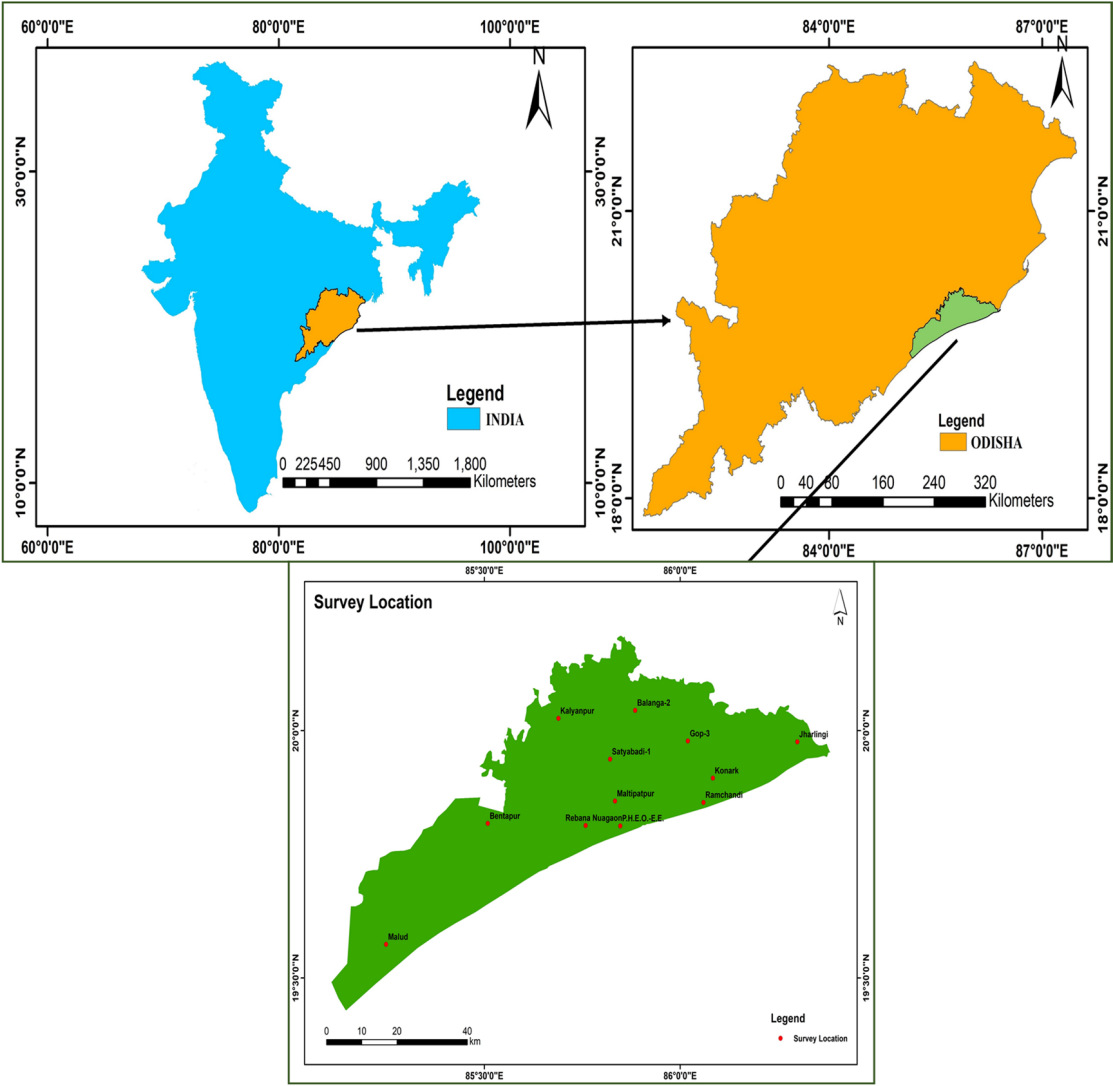
Location map of the study area.

**Fig. 2:**
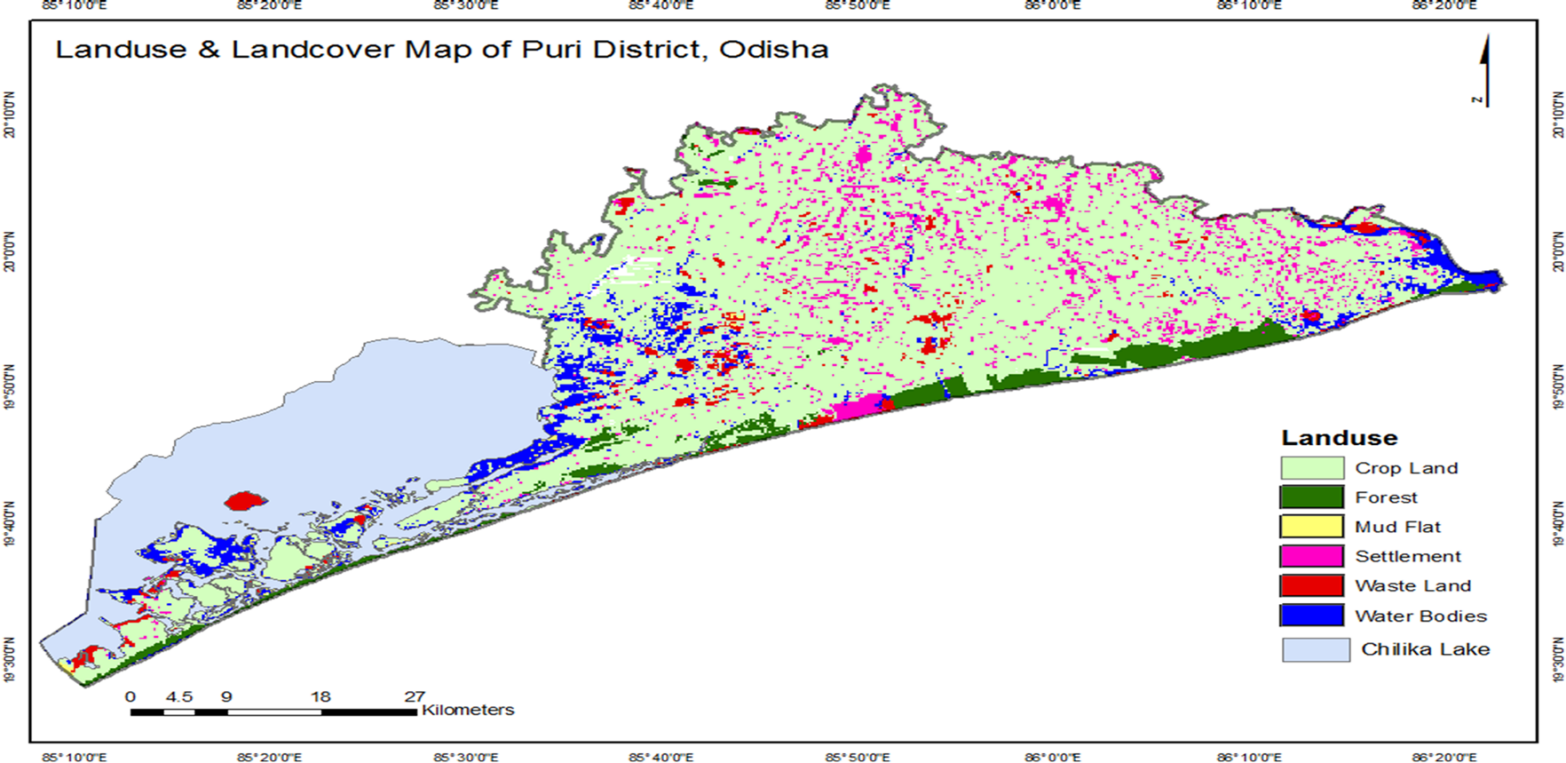
Land use and land cover map of the study area.

Groundwater samples were collected from twelve spatially distributed monitoring locations covering the coastal aquifer system of Puri district. Sampling was conducted during four distinct hydrological seasons, resulting in a total of 47 groundwater samples, as some monitoring wells were seasonally inaccessible. The sampling location map, hydrogeological setting, and drainage network of the study area are presented in Figs. 1 and 2, which illustrate the aquifer characteristics, land use patterns, and surface-water interactions within the district.

### Land use and cropping pattern

The district has geological formations ranging from Archean to Quaternary, with tertiary and quaternary formations covering most of the territory. Quaternary sediments are mainly derived from laterites and alluvium. The district covers 0.35 Mha, with 0.18 Mha net sown and 0.28 Mha grossly cropped. Pipili has the highest cropping intensity (194%), followed by Kakatpur block (193%). Other blocks have varying cropping intensities^19^.

### Collection and analyses of water samples

The study investigated groundwater quality in a region, where it is primarily used for drinking and domestic purposes. Twelve locations were chosen for four seasons, covering the entire region. Water samples were collected from the coastal aquifer of Puri district and analysed for electrical conductivity, pH, total dissolved solid, total hardness, calcium, magnesium, sodium, potassium, nitrate, carbonate, fluoride, and bicarbonate. Instruments used for these measurements were Hanna’s pH meter and EC meter, calibrated with standard solutions. The pH was measured in the field using a hand-held pH meter, and the electrical conductivity was measured in-situ using the pH cum EC meter. Total Dissolved Solids (TDS) in mg/l was determined using the formula TDS = 0.64* Electrical Conductivity in µS. The groundwater sample analysis also included ion concentrations of Na^+^, K^+^, Ca^2+^, Mg^2+^, SO_4_^2−^, NO_3_^−^, Cl^−^, F-, HCO_3_^−^, and TH. Ionic concentrations of cations were analysed using flame photometers, classical volumetric methods, and Spectro-photometers. Nitrate and fluoride were analysed using ion electrodes.

### Calculation of WQI

WQI indicates the overall quality of water for public or intended use. The weight and relative weight for each parameter^20^ are depicted in Table 1. It is calculated by using ten parameters such as pH, Electrical Conductivity, Total hardness, TDS, Bicarbonate, Chloride, Sulphate, Calcium, Magnesium, and Sodium. Horton (1965) used four steps for calculation. The quality parameters are assigned to weights based on their risk factors: TDS and NO_3_^−^ were assigned as 5, pH, EC, SO_4_^2−^ and F^−^ were assigned as 4, 3 for HCO_3_^−^, Cl^−^are assigned as 3; while Na^+^, K^+^, Ca^2+^, TH are assigned as 2, and Mg^2+^is assigned as 1. WQI values can be obtained by computing the following parameters: relative weight (W_i_), quality rating scale (q_i_), and sub-index (SI_i_)^21^.

$$\:{\mathrm{W}}_{\mathrm{i}}=\frac{{\mathrm{w}}_{\mathrm{i}}}{\sum_{\mathrm{i=1}}^{\mathrm{n}}{\mathrm{w}}_{\mathrm{i}}}$$

$$\:\mathrm{q}_{\mathrm{i}} =\frac{{\mathrm{C}}_{\mathrm{i}}}{{\mathrm{s}}_{\mathrm{i}}}\times 100$$

$$\:\mathrm{S}\mathrm{l}\mathrm{i}\:=\mathrm{W}\mathrm{i}\mathrm{*}{\mathrm{q}}_{\mathrm{i}}$$

where, W_i_ is the relative weight, w_i_ is the weight of each parameter, n is the number of parameters, q_i_ is the quality rating scale, C_i_ is the concentrations of each ion, S_i_ is the standard for each parameter, and Sl_i_ is the sub-index for each ion. The WQI is the total of all sub-indices derived for each parameter of a sample and is given by the formula,

$$\:\mathrm{W}\mathrm{Q}\mathrm{I}\:=\:\sum\:_{\mathrm{i=1}}^{\mathrm{n}}{\mathrm{Sl}}_{\mathrm{i}}$$

**Table 1:** Weight and relative weight for each parameter^20^.

S. no.	Chemical parameters	Standard	Weight (w_i_)	Relative weight (W_i_)
1.	pH	8.5	4	0.1
2.	F^−^	1.5	3	0.075
3.	Total hardness	300	2	0.05
4.	TDS	500	5	0.125
5.	HCO_3_^−^	600	1	0.025
6.	Cl^−^	250	5	0.125
7.	SO_4_2-	250	4	0.1
8.	Ca2+	75	2	0.05
9.	Mg2+	50	2	0.05
10.	Na^+^	200	5	0.125
11.	K^+^	12	2	0.05
12.	NO_3_^−^	45	5	0.125
			∑ w_i_=40	∑W_i_=1

### Hydro-chemical analysis for irrigation purposes

In this hydro-chemical analysis the test for irrigation purposes was conducted to find out whether, it is suitable for all the season or not.

### Kelly’s ratio

Classification of groundwater for the purpose of irrigation can be done on the basis of Kelly’s ratio, where sodium is measured against calcium and magnesium. Kelly’s ratio is calculated by using the following formula. KI = Na^+^/ (Ca^2+^ + Mg^2+^) (where all ionic concentrations are expressed in meq/l).

As per this ratio, a value less than 1 for any groundwater sample is considered suitable for irrigation, whereas those with higher than 1 indicate an excess level of sodium and are thus considered unsuitable for irrigation^22^.

### Sodium adsorption ratio (SAR)

Problems with permeability can be caused by sodium in irrigation water. The Sodium Adsorption Ratio (SAR) is evaluated to get around this. It is used to forecast the possibility that sodium could build up in the soil as a result of continuing usage of sodic water^23^. Clay particles in water with a high sodium concentration swap the ion for calcium and magnesium, lowering the soil’s permeability. These soils are often hard when dry and have restricted air and water flow. The SAR is calculated using the following formula,

$$\:\mathrm{S}\mathrm{A}\mathrm{R}=\:[\mathrm{N}\mathrm{a}^{2+}/\sqrt[\mathrm{2}]{{{\mathrm{Ca}}^{\mathrm{2+}}+{\mathrm{Mg}}^{\mathrm{2}}}^{\mathrm{0.5}}}]$$

where, concentration is expressed in meq/l.

### Data analysis

Central Groundwater Board (CGWB) data for Puri district for the year 2021-22 has been analyzed for drinking and irrigation purposes; also, a location map is drawn through ArcGIS software (Arc Map 10.4.1), and a map of land use and land cover (LULC) has been developed utilizing data from the Sentinel-2 satellite imagery dataset provided by the European Space Agency (ESA). The spatial distribution maps of various ions pH, EC, TDS, Na^+^, K^+^, Ca^2+^, Mg^2+^, SO_4_^2-^, NO_3_^-^, Cl^-^, F^-^, HCO^3-^, and WQI have been prepared using ArcGIS software (Arc Map 10.4.1) using the interpolation method (IDW). Irrigation water quality has been analysed by USSL Diagram which is done through AquaChem (version 10.0) software.

## Results and discussions

Groundwater quality refers to the chemical; physical and biological characteristics of water found beneath the earth’s surface in aquifers and underground reservoirs. It is a critical aspect of our water resources, as millions of people around the world rely on groundwater for drinking water, agricultural irrigation and industrial purposes. Ensuring good groundwater quality is necessary for safeguarding public health and the environment. The ideal water fit for drinking or agricultural purposes needs to have some integrity with respect to its dissolved constituents. Since the composition of water is subject to change, monitoring the water quality by comparing it with the international (WHO) and national (BIS) standards is a necessity, which is shown in Table 2.

**Table 2:** Statistical summary of groundwater quality with WHO and BIS standards^24^.

Parameters	Winter				Pre-monsoon				Monsoon				Post-monsoon				BIS (2012)		WHO (2022)
	Min	Max	Mean	SD	Min	Max	Mean	SD	Min	Max	Mean	SD	Min	Max	Mean	SD	Desired	Permissible	Limit
pH	7.2	8.1	7.6	0.3	6.7	8.1	7.5	0.5	7.3	8.5	7.8	0.4	7.3	8.1	7.7	0.3	7.0-8.5	6.5–9.2	6.5–8.5
EC (µS/cm)	234	3246	1086.0	809.2	398.0	17,028	2724.3	4813.8	299	3374	1099.9	961.5	338.0	3097.0	976.8	816.3	500	2000.0	1400.0
TDS (mg/l)	182	2345	796.6	589.3	298.0	11,230	1834.5	3169.2	219	2461	782.3	675.4	246	2315.0	706.4	605.3	500	1500.0	300.0-600.0
TH (mg/l)	35	600	253.3	153.0	150.0	3280	571.8	910.7	90	730	274.6	172.9	90.0	625	231.7	155.7	300	600.0	500.0
Ca^2+^(mg/l)	10	162	60.3	38.1	40.0	640	126.4	174.6	26	174	74.0	43.0	14.0	180	59.2	45.2	75	200.0	40–80
Mg^2+^ (mg/l))	2.4	69.3	24.9	20.2	12.2	408.2	62.2	116.1	4.9	71.7	21.8	18.7	7.3	42.5	20.4	14.5	30	100.0	150.0
Na^+^ (mg/l)	8.7	328	117.1	103.3	15.1	2400	333.1	696.9	13.9	430	101.5	126.7	16.3	325	91.9	100.5	-	200.0	200.0
K^+^ (mg/l)	4.2	235	39.8	65.7	3.8	230	47.6	65.7	3.3	270	44.7	75.0	3.8	274	40.8	74.7	12	-	-
Cl^−^ (mg/l)	10.6	432.5	136.2	120.7	42.5	5374.2	678.7	1568.2	17.7	613.3	158.3	183.2	14.2	432.5	124.1	129.5	200	1000.0	250.0
SO_4_^2−^ (mg/l)	0.0	264.2	71.9	84.7	7.1	465.3	67.6	133.6	4.3	284.2	42.7	77.4	2.4	130.9	24.2	36.2	150	400.0	400.0
HCO_3_^−^ (mg/l)	103.7	762.5	306.3	189.1	152.5	841.8	328.8	199.1	97.6	780.8	288.2	192.4	67.1	884.5	308.1	221.6	-	-	100.0
F^−^ (mg/l)	0.2	2	0.5	0.5	0.1	1	0.5	0.4	0.0	0.7	0.2	0.3	0.0	1.0	0.2	0.4	0.6–1.2	1.2	1.5
NO_3_^−^ (mg/l)	0.7	63.7	15.2	17.6	0.2	97.2	18.9	30.3	1.4	123.1	30.4	39.4	0.0	123.1	18.8	33.6	45.0	–	45.0

### Evaluation of physico-chemical parameters

The study analyzes the chemical parameters of groundwater, including pH, electrical conductivity, TDS, TH, and anions and cations. These parameters determine the ionic composition of the water, allowing for the characterization and description of its quality (USGS)^25^.

### pH

The concentration of hydrogen ions (H⁺) in an aqueous solution is measured by pH, which is the negative logarithm of the H⁺ ion concentration. pH indicates the acidity or alkalinity of a solution. A lower pH means higher acidity, while a higher pH indicates alkalinity. The pH scale ranges from 1 to 14, with 7 being neutral. Values below 7 indicate acidity, and values above 7 signify basicity. In nature, no solution has a pH exactly equal to 7. The limit of pH value for drinking water is specified as 6.5–8.5 (WHO 2004; ISI 2012) with a mean value of 7.6 ± 0.3 for Winter, 7.5 ± 0.5 for Pre-monsoon, 7.8 ± 0.4 for Monsoon and 7.7± 0.3 for post-monsoon seasons. In the study area, the pH ranges from 6.7 to 8.1 for the winter, pre-monsoon, monsoon and post-monsoon season. Overally 47 samples from four respective seasons (PRM, MON, Winter, POM) 6 sample’s pH is higher than 8.0, rest are within 6.7 to 8. Recent regional studies^26,27^ highlight similar pH fluctuations in Eastern Indian coastal aquifers, emphasizing seasonal buffering capacity.

### Electrical conductivity (EC)

Electrical conductivity (EC) is the ability of a substance to conduct or transport electricity. Pure water is a poor conductor of electricity. EC increases as ion concentration in aqueous solutions increases. In examined samples, EC ranges from 234 to 17,028 µS/cm with a mean value of 1086 µS/cm for Winter, 2724.3 µS/cm for Pre-monsoon, 1100 µS/cm for Monsoon, and 976.8 µS/cm for post-monsoon seasons. Three samples crossed the Indian standard limit (2012) and seven crossed the WHO (2021) standard for all four seasons. This aligns with findings of some researcher^28^ who reported comparable EC spikes during pre-monsoon seasons in Odisha’s coastal aquifers. Electrical conductivity can be classified as Type-I (EC<1500µS/cm) if salt enrichment is low, Type-II (1500–3000 µS/cm) if it is medium, and Type-III (EC > 3000 µS/cm) if it is high.

### Total dissolved solids (TDS)

Water is a universal solvent that easily absorbs contaminants. The major components include anions like carbonate, bicarbonate, chloride, sulphate, nitrate and cations such as calcium, magnesium, sodium and potassium. It provides a qualitative measurement of the number of dissolved ions but offers no clear vision of how the ions are related. Milligrams per liter (mg/l) are used to measure it. It represents the general kind, salinity and degree of contamination of groundwater (Table 3).

**Table 3:** USGS classification of groundwater on the basis of TDS.

Type	TDS (mg/l)	No. of sample
Non-saline	< 1000	38
Slightly saline	1000–3000	7
Moderately saline	3000–10,000	1
Highly saline	10,000–35,000	1
Brine	> 35,000	0

The presence of dissolved solids in water has a direct effect on its taste as it may turn the water salty or even brackish. The palatability of drinking water on the basis of total dissolved solid is presented in Table 4.

**Table 4:** Palatability of groundwater on the basis of TDS^29^.

TDS (mg/l)	Palatability	No. of samples
< 300	Excellent	3
300–600	Good	14
600–900	Fair	19
900–1200	Poor	2
> 1200	Unacceptable	9

The TDS of the groundwater of the study area varies from 182 mg/l to 11,230 mg/l for four seasons, with a mean value of 589.3 ± 298 for winter, 1834.5 ± 3169.2 for pre-monsoon, 782.3 ± 675.4 for monsoon and 706.4 ± 605.3 for post-monsoon seasons. These findings approve with one of the researchers^30^, who classified 75% of groundwater samples in nearby Ganjam district as non-saline with fair palatability during monsoon. From the above tables, it is evident that the groundwater of the study area is non-saline and fair to good in terms of palatability.

### Total hardness (TH)

Water hardness is traditionally measured by its ability to precipitate soap and is mainly caused by soluble, divalent metallic cations from natural geogenic sources. The primary contributors are calcium, derived from limestone, and magnesium, from dolomite or other magnesium-bearing minerals. Groundwater tends to be harder than surface water due to prolonged contact with geological formations. The TH of the study area was found to differ from 35 to 3280 mg/l with a mean value of 153±150 for winter, 571.8± 910.7 for pre-monsoon, 730± 274.6 for monsoon and 231.7± 155.7 for post-monsoon seasons. Water can be classified as soft or hard on the basis of its TH value (Table 5).

**Table 5:** Classification of water based on hardness.

Total hardness	Water class	No. of samples
< 75	Soft	1(2%)
75–150	Moderately Hard	9(19%)
150–300	Hard	22(47%)
> 300	Very hard	15(31%)

Source:^31^.

Based on the classification, 2% of samples were found as soft, 19% samples were found as moderately hard, 47% samples were found hard, and 31% samples were very hard. The liable limit of total hardness of water for drinking purposes is 300 to 600 (BIS-IS: 10500:1991) thus, the water in the study area has been found to be hard to very hard, thus, the water in the study area is safe for drinking purposes.

### Major cations (Ca^2+^, Mg^2+^, Na^+^, K^+^)

**Fig. 3:**
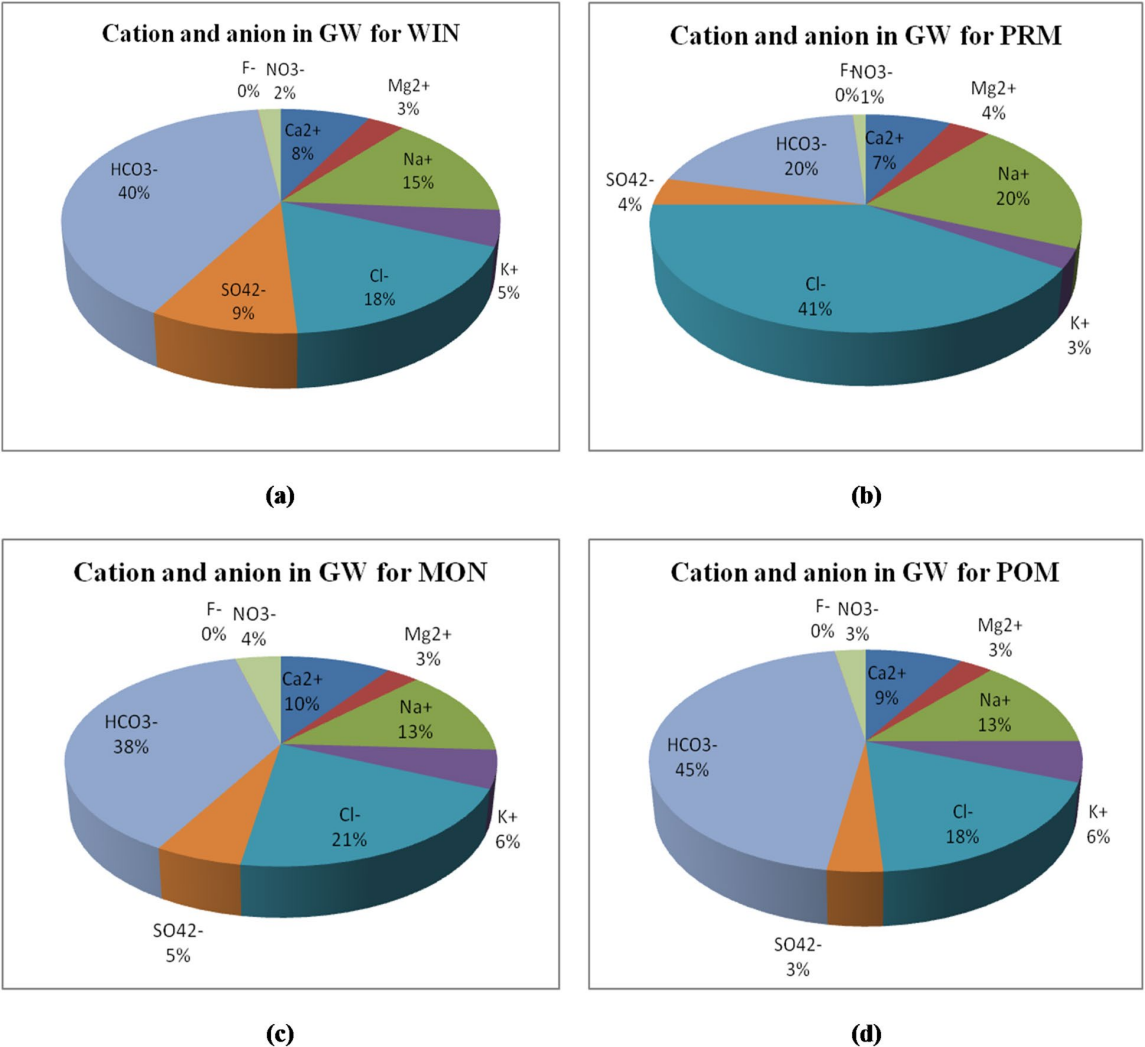
Abundance of cations and anions distribution in groundwater.

Cations are the positively charged ions, which are mostly metallic in nature. Various cations present in groundwater sample include calcium, magnesium, sodium and potassium, where the former two are divalent metallic ions and the latter two are monovalent metallic ions. These are found naturally in groundwater while anthropogenic activities can increase their concentration in the aqueous solution. These are measured in mg/l or meq/l. The abundance of various cations distribution in groundwater is depicted in Fig. 3.

### Calcium (Ca^2+^)

The concentration of calcium in the study area has values in the range from 10 to 640 mg/l with a mean value of 60.3 ± 38.1 for winter, 126.4 ± 174.6 for pre-monsoon, 74 ± 43 for monsoon and 59.2 ± 45.2 for post-monsoon seasons. Out of 47 samples for all four season 35 samples fall below the permitted limit (75 mg/l) of Ca^2+^ as suggested by WHO, (2004) and are safe for drinking purposes. High levels of calcium in water increase its hardness, leading to calcium carbonate deposits in distribution systems and reduced soap effectiveness. Excessive calcium in drinking water is undesirable as it can cause abdominal issues, while insufficient intake is linked to health problems such as osteoporosis, kidney stones, hypertension, and stroke.

### Magnesium (Mg^2+^)

Magnesium content in the study area ranges from 2.4 mg/l to 408 mg/l with a mean value of 24.9 ± 20.2 for winter, 62.2 ± 116.1 for pre-monsoon, 21.8 ± 18.7 for monsoon and 20.4 ± 14.5 for post-monsoon seasons. Out of 47 samples 46 samples fall below the permissible limit of magnesium (100 mg/l ISI, 1993 and 150 mg/l WHO, 2004) and so is safe for drinking purposes. It is one of the four most important metals present in the human body and other three being calcium, potassium and sodium. Deficiency of magnesium can cause many metabolic and nutritional disturbances and also lead to hypocalcaemia. Increased concentration of magnesium in drinking water has the same effects as that of calcium.

### Sodium (Na^+^)

The sodium ion is ubiquitous in water^29^. The main source of sodium in natural water is from the release of the soluble products during the weathering of rocks. The ion exchange between Ca^2+^/ Mg^2+^ from groundwater with Na^+^/K^+^ of the country rock also contributes to the increased concentration of sodium in groundwater. Other sources include saltwater intrusion, infiltration from landfills, etc.

In the study area, the concentration of sodium differs from 8.7 to 2400 mg/l for all the four seasons. With a mean value of 117.1 ± 103.3 for winter, 331.1 ± 696.9 for pre-monsoon, 101.5 ± 126.7 for monsoon and 91.9 ± 100.5 for post-monsoon seasons. Excluding 5 samples, all 42 samples within permissible limit, as recommended by 200 mg/l (WHO, 2004). This is reliable with^32^, who observed that sodium levels remained below WHO limits in > 90% of coastal groundwater wells in Odisha. So, all the 42 groundwater samples are fit for drinking purposes. The World Health Organization (WHO) states that sodium intake varies by age group, can lead to abnormal metabolism, heart attacks, and affect the taste of drinking water.

### Potassium (K^+^)

Potassium is vital in groundwater as it directly impacts drinking water safety. Found as soluble salts, it enters groundwater through rock and mineral weathering. Understanding potassium’s distribution, behaviour, and management is crucial for ensuring clean drinking water, promoting sustainable agriculture, and protecting the environment. The potassium concentration of the study area differs from 3.8 to 274 mg/l. With a mean value of 39.8 ± 65.7 for winter, 47.6 ± 65.7 for pre-monsoon, 44.7 ± 75 for monsoon and 40.8 ± 74.7 for post-monsoon seasons. 31% of the samples surpassed the allowable limit 20 mg/l (WHO 2004) of potassium. There is no adverse effect of high concentration of K^+^ in drinking water as it excretes out easily in the form of sweat. A single heavy dose can lead to chest tightness, nausea, vomiting, hyper kalmia, shortness of breath and heart failure.

### Major anions (SO_4_^2−^, NO_3_^2−^, Cl^−^, F^−^, HCO_3_^−^)

Negatively charged anions are primarily found in groundwater naturally, while anthropogenic activities can increase their concentration. Sulphate (SO_4_^2−^), nitrate (NO_3_^2−^), chloride (Cl^−^), fluoride (F^−^), and bicarbonate (HCO_3_^−^) are the four main anions in groundwater. Below, each of them is further explained. The abundance of various anions distribution in groundwater is depicted in Fig. 3.

### Sulphate (SO_4_^2−^)

Groundwater contains sulphate, a crucial anion involved in geochemical processes and water quality, originating from natural and human-made sources like weathering minerals and rocks. The concentration of sulphate in the study area ranges from 2.4 to 264.2 mg/l for all the four seasons, with a mean value of 71.9 ± 84.7 for winter, 67.6 ± 133.6 for pre-monsoon, 42.7 ± 77.4 for monsoon and 24.2 ± 36.2 for post-monsoon seasons. Maximum (91%) of the groundwater samples from the study area have a value less than the permissible limit of 150 mg/l (WHO 2004). High sulphate groundwater is not approved for drinking since it can irritate the gastrointestinal tract. Also, it could lead to distribution system corrosion.

### Nitrate (NO_3_^−^)

Nitrate concentration in groundwater, measured in ppm, can be harmful to the environment and human health due to factors like agricultural runoff, sewage discharge, and nitrogen-based fertilizers^33^. In the study area, the concentration of nitrate is ranged from 0.2 mg/l to 123 mg/l, with a mean value of 15.2 ± 17.6 for winter, 18.9 ± 30.3 for pre-monsoon, 30.4 ± 39.4 for monsoon and 18.8 ± 33.6 for post-monsoon seasons. The highest permissible limit of nitrate in groundwater for drinking purposes is 45 mg/l. Maximum (95%) of the groundwater samples content is less than the permissible limit. A similar nitrate distribution pattern was observed by^34^ in peri-urban areas of Puri and Cuttack. Nitrate level above the permissible limits known to cause a potentially fatal blood disorder in infants under six months of age known as Blue Baby Syndrome.

### Chloride (Cl^-^)

Seawater is the only significant source of chloride in natural water, while groundwater can also contain chloride due to weathering, sediment leaching, wind-blown salt, saltwater intrusion, residential and industrial waste discharge, and municipal effluents. Chloride in the groundwater sample of the study area ranges from 10.6 to 5374.2 mg/l, with a mean value of 136.2 ± 120.7 for winter, 678.7 ± 1568.2 for pre-monsoon, 158.3± 183.2 for monsoon and 124.1 ± 129.5 for post-monsoon. Except for one sample all samples have less than the permissible limit of chloride in groundwater (250 mg/l ISI, 1000 mg/l WHO). Chloride is non-toxic to humans, but high levels give it a salty taste, making it unusable. Consistent consumption of chlorinated water may lead to bladder cancer^35^.

### Fluoride (F^-^)

Elemental fluoride does not occur in nature due to its reactivity. The weathering of primary rock and leaching of fluoride which contains minerals in soils yield fluoride rich groundwater that is commonly associated with low calcium and high bicarbonate ions. The permitted limit of fluoride in drinking water is 1.5 mg/l according to the WHO, 1984.

The desirable limit of fluoride according to BIS is 0.6 to 1.2 mg/l and the maximum permissible limit in absence of any other source is 1.5 mg/l (ISI,2012). In the study area fluoride content ranges from 0.2 to 2 mg/l, with a mean value of 0.5 ± 0.5 for winter, 1 ± 0.5 for pre-monsoon, 0.7 ± 0.2 for monsoon and 0.2 ± 0.4 for post-monsoon seasons. Low concentration of fluoride in drinking water has an advantageous effect on teeth but excessive exposure can cause mild dental fluorosis or crippling skeletal fluorosis in extreme cases.

### Bicarbonate (HCO_3_^−^)

Monitoring and managing bicarbonate levels in groundwater is crucial for its suitability for various uses, including drinking water and industrial processes, and preserving aquatic ecosystems. High concentrations have no serious health effects but should not exceed 250 mg/l. Understanding bicarbonate distribution is essential. The concentration of bicarbonate in the groundwater sample of the study area ranges from 67.1 to 884.5 mg/l for all the four seasons, with a mean value of 306.3 ± 189.1 for winter, 328.8 ± 199.1 for pre-monsoon, 288.2 ± 192.4 for monsoon and 308.1 ± 221.6 for post-monsoon seasons.

**Fig. 4:**
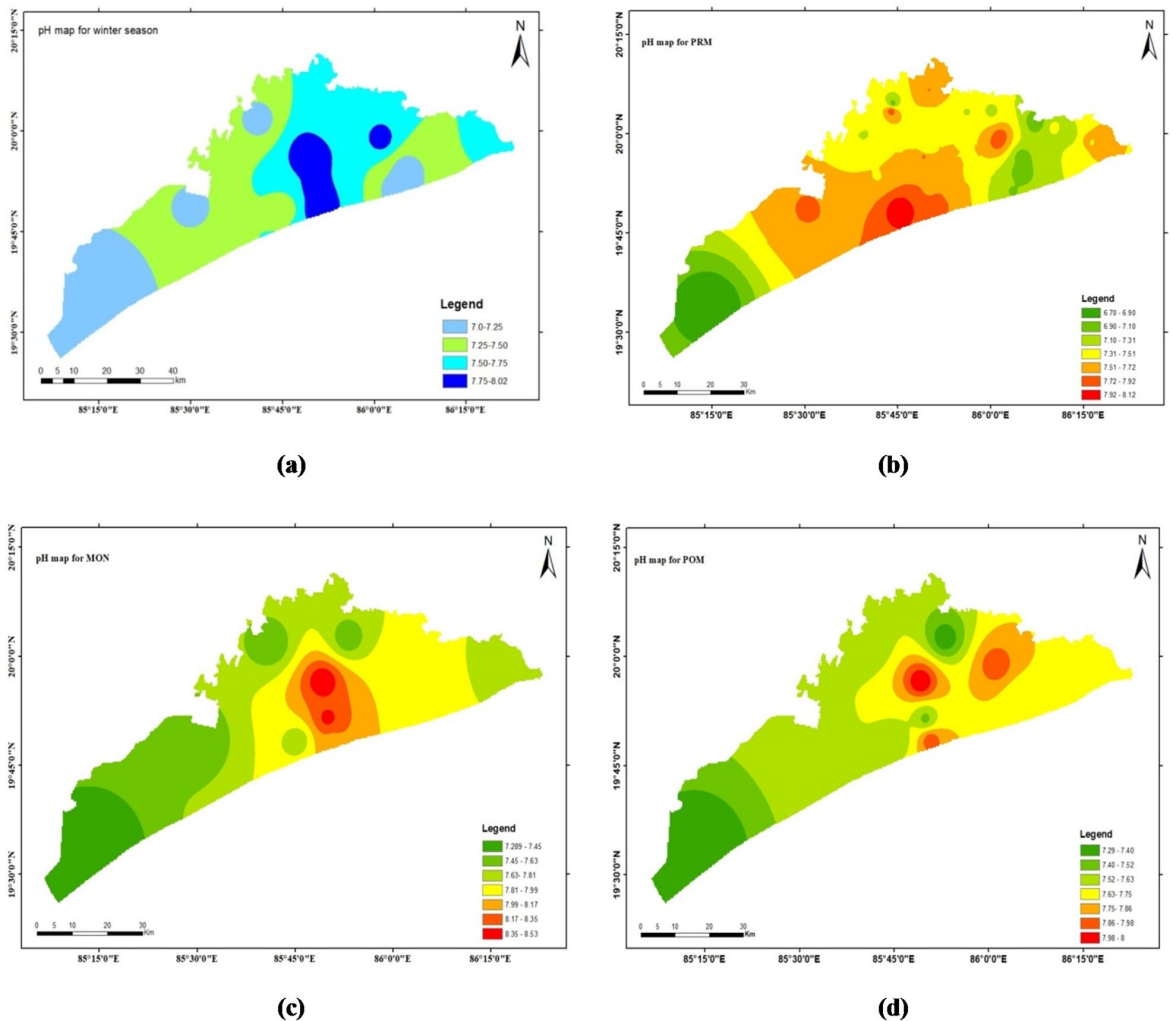
The spatial distribution maps done through ArcGIS software (ArcMap 10.4.1) by interpolation technique (IDW).

**Fig. 5:**
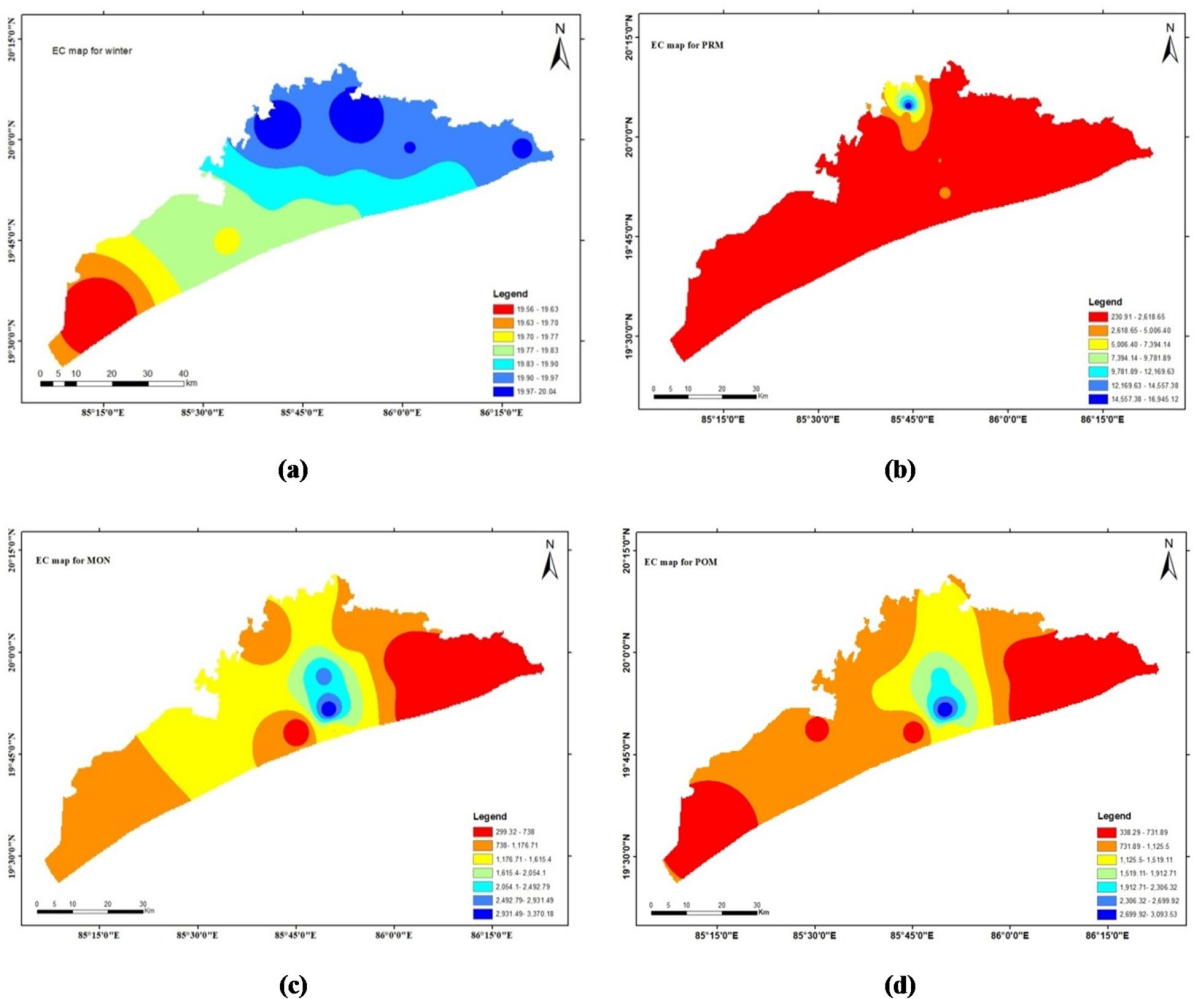
The spatial distribution maps done through ArcGIS software (ArcMap 10.4.1) by interpolation technique (IDW).

**Fig. 6:**
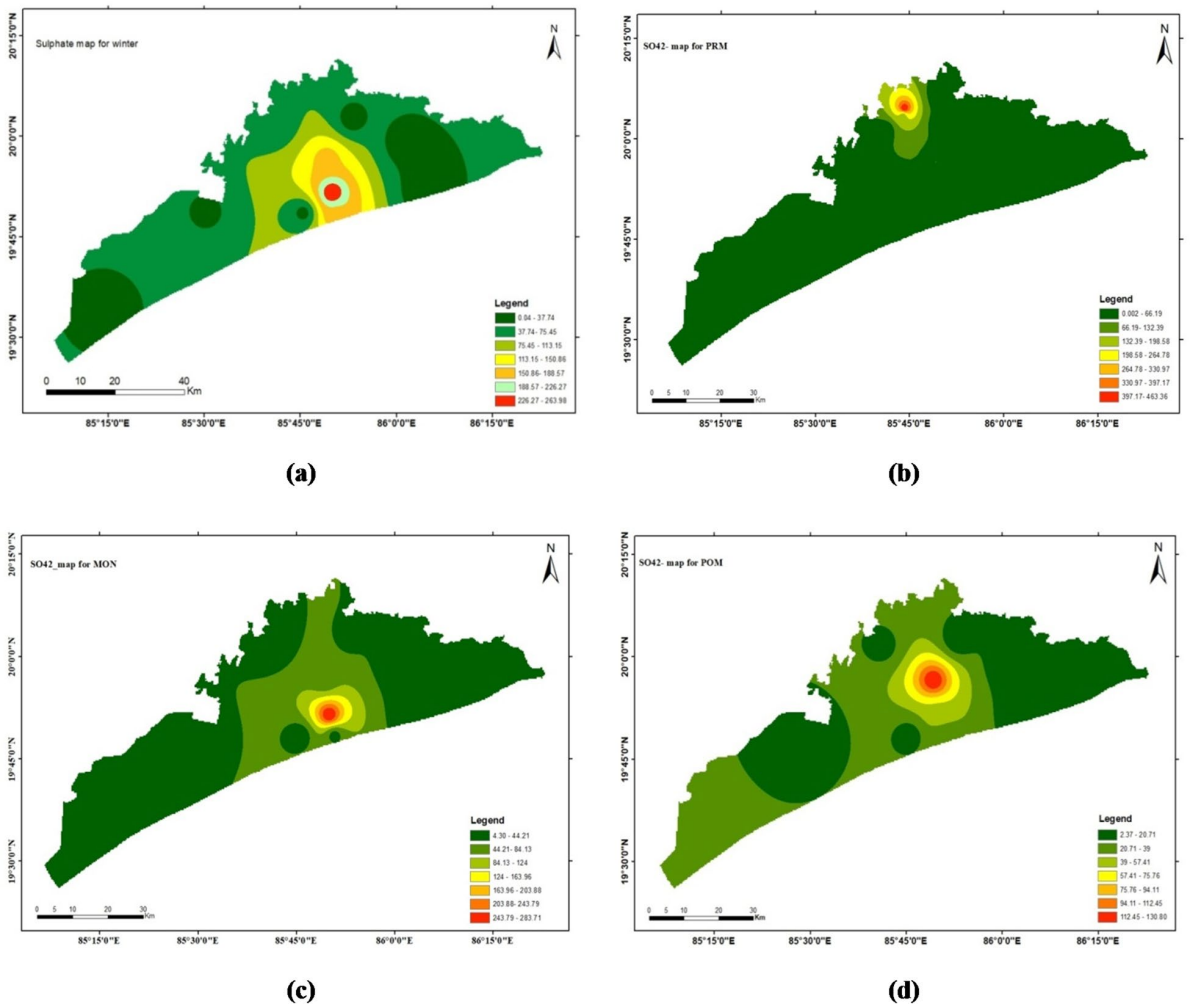
The spatial distribution maps done through ArcGIS software (ArcMap 10.4.1) by interpolation technique (IDW).

**Fig. 7:**
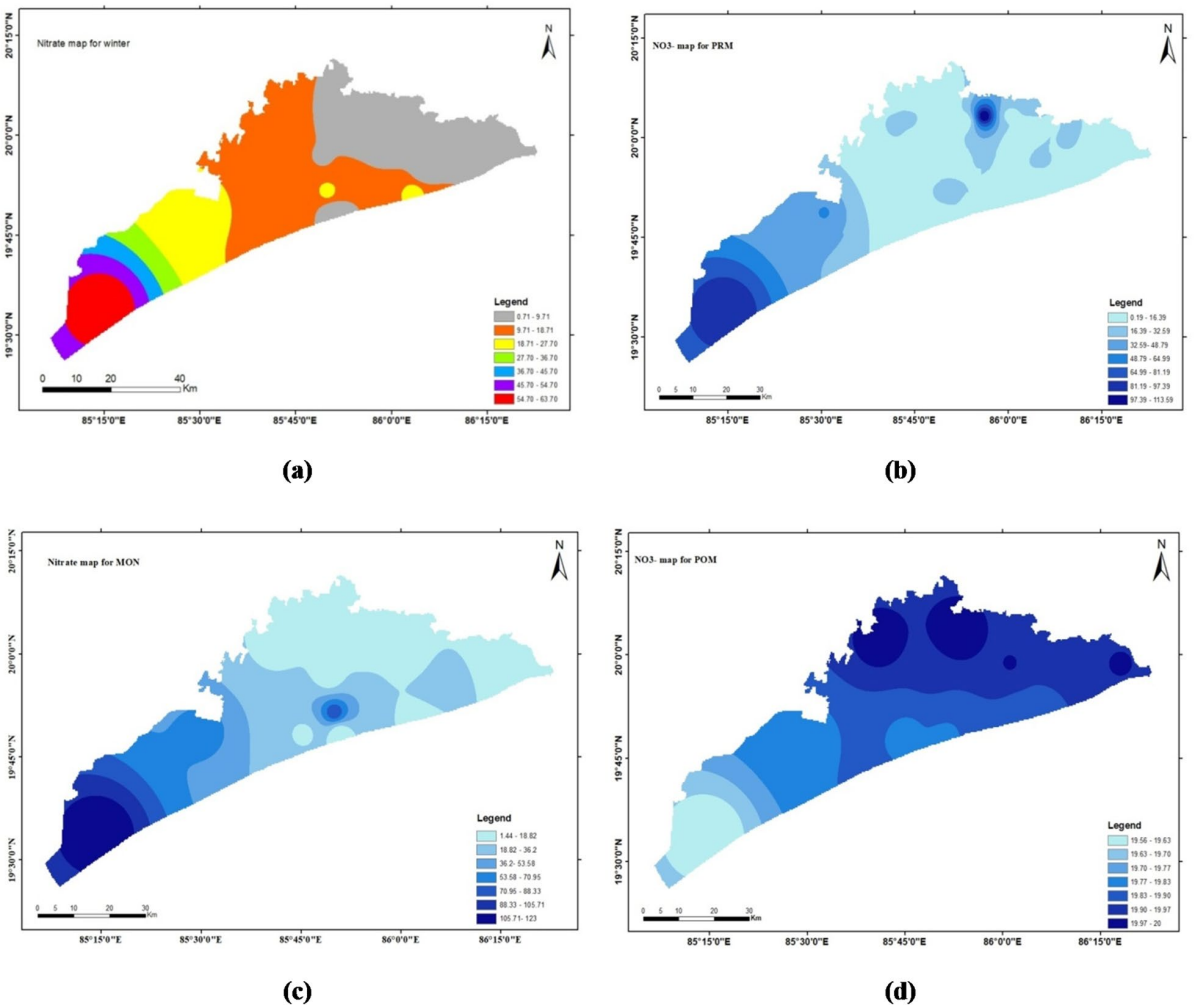
The spatial distribution maps done through ArcGIS software (ArcMap 10.4.1) by interpolation technique (IDW).

The spatial distribution maps done through ArcGIS software (Arc Map 10.4.1) by interpolation technique (IDW) are depicted in Figs. 4, 5, 6 and 7 respectively. This method estimates values at unsampled locations based on the spatial proximity of known data points, assigning greater influence to points closer to the prediction location. The resulting maps provide a visual representation of the variation and concentration of the studied parameters across the geographical area, enabling the identification of spatial trends, hotspots, and zones of interest. These interpolated maps serve as an effective tool for understanding the spatial heterogeneity of the dataset and support informed decision-making in environmental assessment and management.

### Water quality index

**Fig. 8:**
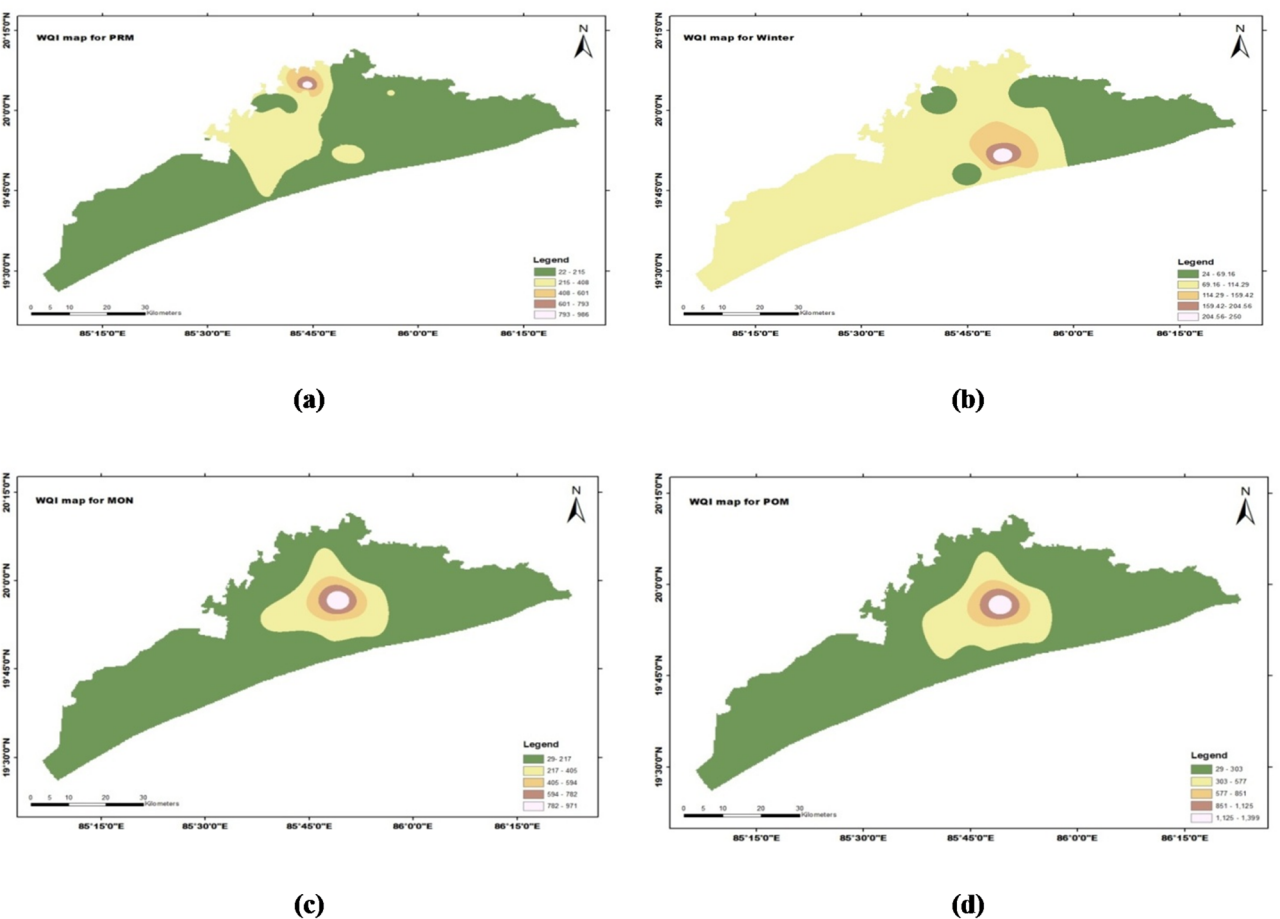
Water quality index maps for all the sampling locations for the winter, pre-monsoon, monsoon and post-monsoon (2020-21).

Water Quality Index for all the sampling locations were calculated for the Winter (Dec-Feb), Pre-monsoon (Mar-May), Monsoon (June-Sep) and Post-monsoon (Oct-Nov) (2020-21), using the equations (From Material and methods) (Fig. 8). The water quality classification based on WQI in Puri district are depicted in Tables 6 and 7 respectively. This shows a variation in water quality across the seasons, winter and post-monsoon seasons having the highest percentage of “Excellent” water quality samples and the monsoon season showing the highest percentage of “Unfit” samples. Similar seasonal WQI trends were reported in the Kendrapara region by^36^, suggesting monsoonal dilution and winter concentration effects. Additionally, the recent work has explored machine learning integration with WQI prediction, offering better resolution of spatial and seasonal variation in aquifer systems^37,38^.

**Table 6:** Water quality classification based on WQI^39^.

Sl. No.	WQI range	Type of water
1	< 50	Excellent
2	50.1 – 100	Good
3	100.1 – 200	Poor
4	200.1 – 300	Very poor
5	> 300.1	Unfit for drinking

**Table 7:** Water quality classification based on WQI value for the year (2021-21) in puri district.

WQI value	Water types	% Samples			
		Winter	PRM	MON	POM
< 50	Excellent	33.3	25.0	30	33
50–100	Good	50.0	33.3	50	25
100–150	Poor	16.7	13.3	8	8
150–300	V.Poor	8.3	6.7	8	8
> 300	Unsuitable	NIL	16.6	17	17

Groundwater quality varies significantly across the study area, as evidenced by the seasonal distribution of Water Quality Index (WQI) values. Winter exhibits the most favorable groundwater quality, with 33.3% of samples classified as excellent (WQI < 50) and 50% categorized as good, indicating minimal contamination and effective dilution conditions. In contrast, the pre-monsoon (PRM) season shows a modest decline in water quality, with only 25% of samples rated as excellent and 33.3% as good, reflecting increased solute concentration resulting from restricted recharge. This seasonal behavior reflects localized recharge effects and dilution in less impacted zones, whereas areas experiencing limited recharge display elevated solute levels.

During the monsoon season, groundwater quality exhibits pronounced spatial variability. Although the percentage of unsuitable samples (WQI > 300) increases to 17% due to surface runoff and pollutant influx, 30% of samples still fall within the excellent category and 50% remain satisfactory, suggesting that recharge-driven dilution continues to benefit certain locations. The observed variability during monsoon highlights the combined influence of hydrological processes and land-use interactions, where recharge, runoff, and anthropogenic inputs collectively control groundwater quality. Post-monsoon (POM) conditions indicate a partial recovery in groundwater quality, with 33% of samples classified as excellent and a reduction in the proportion of good and poor categories. Nevertheless, 17% of samples remain unsafe for drinking, implying that residual contamination persists in specific locations even after monsoonal flushing. Across all seasons, the poor (WQI 100–150) and very poor (WQI 150–300) categories consistently remain below 17%, indicating that severe groundwater quality degradation is confined to localized zones rather than being widespread.

Overall, the WQI analysis demonstrates that groundwater quality tends to deteriorate during pre-monsoon and monsoon periods due to hydrological stress and human influences, while improved conditions are generally observed during winter and post-monsoon seasons, emphasizing the strong role of seasonal hydrology and land-use practices in shaping groundwater quality dynamics^40^.

### Groundwater suitability for irrigation purposes

Electrical conductivity (EC) and total dissolved solids (TDS).

Salinity of water is a significant problem when it comes to irrigation water quality. The amount of salt present in the water can be measured by measuring the Total Dissolved Solids or Electrical Conductivity, but they do not provide any information about the type of salt present. Water quality irrigation on the basis of TDS and EC is presented in Table 8.

**Table 8:** Irrigation water quality on the basis of TDS and EC.

TDS (ppm)	EC (µS/cm)	% of samples				Salinity hazard
		WIN	PRM	MON	POM	
< 500	< 800	42	25	42	59	Low
500–1000	800–1600	42	42	42	25	Medium
1000–2000	1600–3000	8	8	8	8	High
> 2000	> 30,000	8	25	8	8	V.High

From the Table 8, on the basis of EC and TDS the groundwater samples are found as salinity hazard is low to medium, so it is suitable for irrigation purposes in that region.

### Kelly’s ratio

Classification of groundwater for the purpose of irrigation can be done on the basis of Kelly’s ratio, where sodium is measured against calcium and magnesium. Kelly’s ratio is calculated using the following formula. As per this ratio, a value less than 1 for any groundwater sample is considered as suitable for irrigation whereas those higher than 1 indicate an excess level of sodium and are thus considered unsuitable for irrigation.

In the study area, groundwater suitability for irrigation varies across seasons. In the winter, 41% of samples are suitable, while 59% are unsuitable. For the pre-monsoon season, 52% are suitable and 48% unsuitable. During the monsoon, 84% are suitable and 16% unsuitable. In the post-monsoon season, 67% are suitable and 33% are unsuitable for irrigation.

### Sodium adsorption ratio (SAR)

The Sodium Adsorption Ratio (SAR) is used to predict the potential for sodium accumulation in soil due to the high concentration of sodium in irrigation water. This results in clay particles exchanging sodium for calcium and magnesium, reducing soil permeability and causing restricted air and water circulation^41^. Classification on the basis of SAR values along with the respective number of samples in the category concerned are presented in Table 9.

**Table 9:** Classification of ground water based on SAR^42^.

SAR	Water quality	% of samples			
		WIN	PRM	MON	POM
< 10	Excellent	41	67.64	83.33	75
10–18	Good	33.33	24.68	8.33	16.66
18–26	Doubtful	8.33	3.22	0	0
> 26	Unsuitable	16.66	3.22	8.34	8.33

From the above table, it can be observed that the percentage of groundwater samples classified as excellent based on SAR values is high across all four seasons considered in this study. Among these, the pre-monsoon (PRM) season exhibits the best overall water quality, with the highest proportion of samples falling under the “Excellent” category. This condition occurs because groundwater during the pre-monsoon period is relatively less diluted, and ions such as sodium may remain lower due to minimal recharge prior to the onset of monsoonal rainfall. Consequently, the higher proportion of excellent SAR values during the pre-monsoon season (67.64%) can be attributed to reduced dilution variability and lower sodium mobilization before monsoonal recharge processes begin. In contrast, winter season SAR values show a comparatively lower proportion of excellent samples (41%), which reflects the accumulation of residual salts following post-monsoon evapotranspiration and reduced groundwater movement during this period. These seasonal variations in SAR values highlight the influence of hydrological processes on sodium hazard in groundwater.

**Fig. 9:**
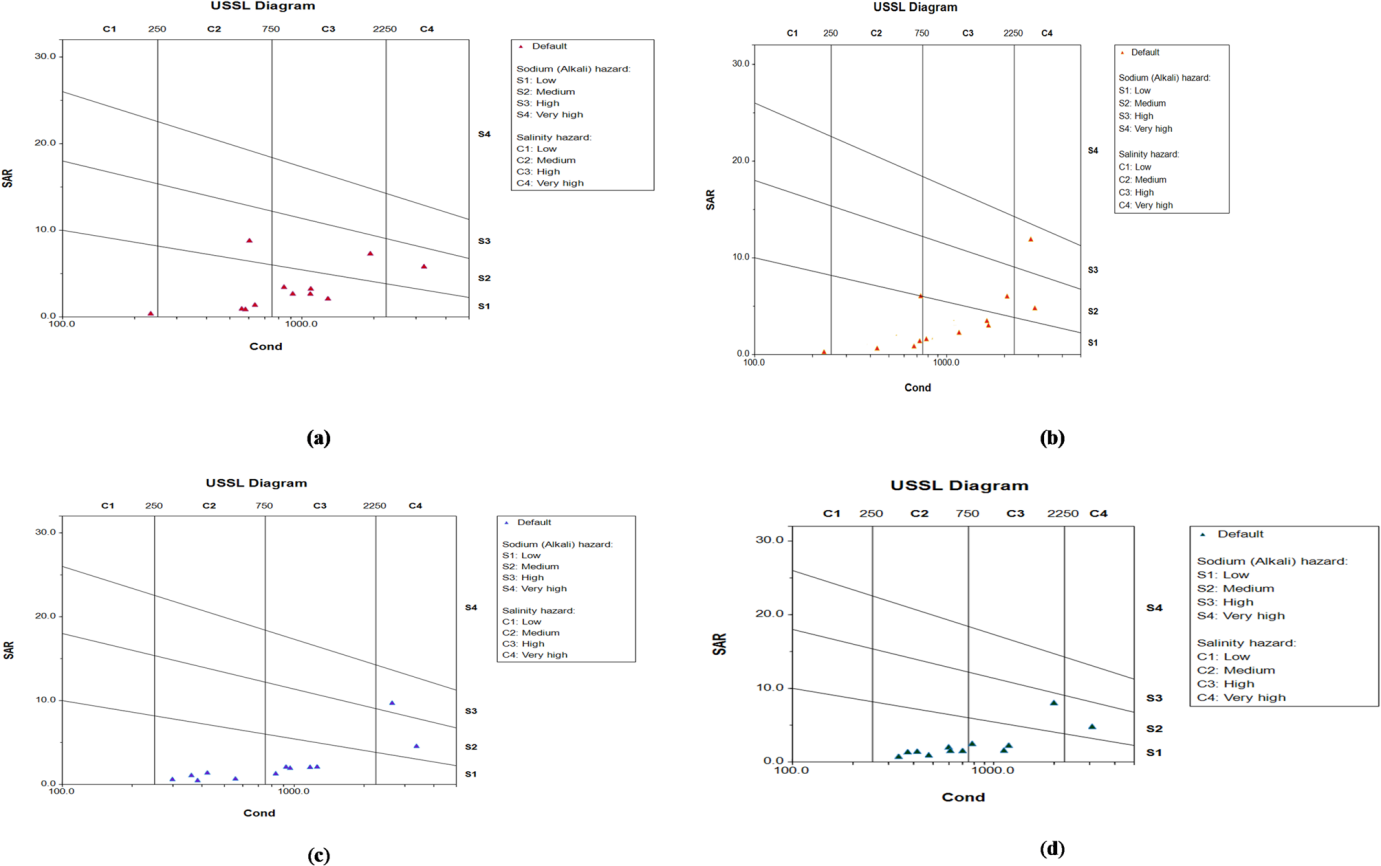
US salinity diagram (USSL) for all the four seasons (winter, pre-monsoon, monsoon, post-monsoon).

This interpretation is further supported by the United States Salinity Laboratory (USSL)^43,44^, diagrams prepared for all four seasons winter, pre-monsoon, monsoon, and post-monsoon using AquaChem software. The seasonal distribution of groundwater samples across different salinity and sodium hazard classes is clearly represented in the USSL diagram (Fig. 9), which corroborates the SAR-based assessment of irrigation water quality.

From the USSL diagram Fig. 9(a), representing the winter season, it is observed that one groundwater sample each falls within the C1S1 (low salinity–low sodium hazard), C2S2 (medium salinity–medium sodium hazard), and C3S2 (high salinity high sodium hazard) classes. Additionally, three samples are plotted in the C2S1 (medium salinity low sodium hazard) category, while four samples fall under the C3S1 (high salinity–low sodium hazard) class. From the second USSL diagram, Fig. 9(b), it is noted that the higher number of plotted samples in the pre-monsoon USSL diagram reflects the inclusion of additional operational monitoring well available only during that season. The groundwater samples for the pre-monsoon season show that one sample each falls in the C1S1 (low salinity low sodium hazard) and C4S3 (very high salinity high sodium hazard) categories. Furthermore, two samples each are classified under C3S2 (high salinity high sodium hazard) and C4S2 (very high salinity medium sodium hazard), while the maximum number of groundwater samples are observed in the C2S1 (medium salinity–low sodium hazard) and C3S1 (high salinity–low sodium hazard) categories. From the third USSL diagram, Fig. 9(c), corresponding to the monsoon season, one sample each falls under the C4S3 (very high salinity–high sodium hazard) and C4S2 (very high salinity–medium sodium hazard) classes. In contrast, five groundwater samples each are plotted within the C2S1 (medium salinity–low sodium hazard) and C3S1 (high salinity–low sodium hazard) categories. From the fourth USSL diagram, Fig. 9(d), representing the post-monsoon season, one groundwater samples each falls in the C3S2 (high salinity–high sodium hazard) and C4S2 (very high salinity–medium sodium hazard) classes. The remaining samples are primarily distributed within the C2S1 (medium salinity–low sodium hazard) and C3S1 (high salinity–low sodium hazard) categories, comprising seven and three samples, respectively.

## Conclusions

Groundwater continues to serve as a critical resource for sustaining human development, ecological balance, and climate-resilient water security, particularly in coastal regions like the Puri district. The present study provides a comprehensive seasonal assessment of groundwater quality by analyzing major physicochemical parameters, including EC, pH, TDS, total hardness, Ca²⁺, Mg²⁺, Na⁺, K⁺, NO₃⁻, CO₃²⁻, HCO₃⁻, and F⁻, over four distinct seasons winter, pre-monsoon, monsoon, and post-monsoon. These results were evaluated against BIS (2012) and WHO (2022) drinking water standards to assess potability, while irrigation suitability was examined using key indices such as Kelly’s Ratio, EC, TH, and SAR. The Water Quality Index (WQI) analysis indicates that a majority of groundwater samples across all seasons fall within the good to excellent categories, suggesting that, in general, the groundwater is suitable for drinking^45^. Seasonal WQI patterns reveal that 50% and 33.3% of samples in winter; 33.3% and 30% in pre-monsoon; 50% and 25% in monsoon; and 25% and 33% in post-monsoon are classified as good and excellent, respectively, highlighting overall acceptable water quality with minor spatial deterioration. Nevertheless, isolated pockets, particularly in areas such as Kalyanpur and Maltipatpur, exhibit elevated EC and TDS, indicating localized salinity and potential seawater intrusion risks. These anomalies underscore the importance of site-specific monitoring and targeted interventions. The irrigation suitability assessment demonstrates that the groundwater is generally appropriate for agricultural use. Kelly’s Ratio values in the monsoon and post-monsoon seasons reflect favorable conditions, while SAR values throughout all seasons consistently classify the water as excellent to good, corroborating findings from previous coastal aquifer studies that report low sodium hazard when supported by managed recharge and proper crop management practices. Although most samples remain below critical limits for salinity and sodium concentrations, the presence of isolated high-salinity pockets warrants caution to prevent long-term soil degradation and reduced crop productivity. The hydrogeochemical analysis suggests that seasonal variations in groundwater quality are primarily influenced by recharge processes, water–rock interactions, ion exchange mechanisms, and potential seawater intrusion in low-lying coastal zones. Elevated parameters such as EC, TDS, and TH in specific locations reflect localized anthropogenic and natural impacts, highlighting the need for continuous monitoring to track temporal trends and emerging water quality risks. In conclusion, while groundwater in the Puri district is largely suitable for drinking and irrigation, localized salinity and seasonal fluctuations highlight the need for proactive management. Although most samples meet drinking and irrigation standards, isolated high-salinity pockets highlight the need for continuous monitoring, managed aquifer recharge, and sustainable irrigation practices. Implementing the above recommendations will enhance groundwater sustainability, support agricultural productivity, and contribute to climate-resilient water security in this vulnerable coastal region. The findings directly support SDG-6 by improving drinking water safety assessment and SDG-13 by strengthening climate-resilient groundwater management strategies in vulnerable coastal regions.

Based on the findings, the study recommends several management measures to ensure long-term water security:

1. **Regular groundwater monitoring**: Establish a systematic groundwater quality monitoring network to detect seasonal and spatial variations, particularly in salinity-prone areas.
2. **Managed aquifer recharge (mar)**: Implement MAR strategies, including rainwater harvesting and artificial recharge structures, to dilute high-salinity pockets and maintain sustainable groundwater levels.
3. **Sustainable irrigation practices**: Promote crop rotation, soil salinity management, and efficient irrigation techniques (e.g., drip or sprinkler irrigation) to minimize sodium and salinity hazards.
4. **Targeted remediation**: Identify and prioritize remediation in high-risk areas with elevated EC and TDS to prevent adverse health and agricultural impacts.
5. **Integration with policy and community awareness**: Encourage local authorities and communities to adopt water conservation measures, raise awareness of groundwater risks, and integrate scientific findings into district-level water management planning.

## Supplementary Information

Below is the link to the electronic supplementary material.

Supplementary Material 1

## Data Availability

All data used in the study is taken in the research field and the geological data is available in public domain of Central Groundwater Board. The datasets used and/or analyzed during the current study are available from the corresponding author on reasonable request.
