# Supplementary material for: Seasonal groundwater quality assessment and irrigation suitability in coastal aquifers of Puri District, Odisha, India
Source: Sci Rep. 2026 Feb 9;16:7895. doi: 10.1038/s41598-026-39145-8 (PMC12953622; doi:10.1038/s41598-026-39145-8)
Supplement: Supplementary file 1 — Supplementary Material 1 [file 41598_2026_39145_MOESM1_ESM.docx]

**Supplementary Material**

**Appendix-A Physiochemical parameters in groundwater for winter season (Raw data, Supplementary Material)**

| **District** | **Location** | **pH** | **EC** | **TDS** | **TA** | **TH** | **Ca^2+^** | **Mg^2+^** | **Na^+^** | **K^+^** | **Fe^2+^** | **Cl^-^** | **SO_4_^2-^** | **HCO_3_^-^** | **F^-^** | **NO_3_^-^** |
| --- | --- | --- | --- | --- | --- | --- | --- | --- | --- | --- | --- | --- | --- | --- | --- | --- |
| **Puri**  **(WINTER)** | Balanga-2 | 7.6 | 1092 | 842 | 390 | 285 | 68 | 27.9 | 127 | 4.2 | 1.6 | 95.7 | 20.2 | 475.8 | 0.2 | 1.9 |
|  | Bentapur | 7.3 | 1087 | 822 | 280 | 240 | 84 | 7.3 | 95.7 | 89.1 | 0.1 | 141.8 | 31.0 | 341.6 | 0.7 | 21.7 |
|  | Gop-3 | 7.9 | 605 | 438 | 150 | 35 | 10 | 2.4 | 120 | 10.4 | 0.3 | 85.1 | 12.4 | 183.0 | 0.4 | 4.8 |
|  | Jharlingi | 7.6 | 844 | 604 | 180 | 180 | 52 | 12.2 | 107 | 10.2 | 0.8 | 113.4 | 66.1 | 219.6 | 0.3 | 1.9 |
|  | Kalyanpur | 7.3 | 918 | 690 | 240 | 250 | 70 | 18.2 | 98 | 14 | 0.2 | 106.4 | 43.3 | 292.8 | 0.2 | 18.0 |
|  | Konark | 7.3 | 234 | 182 | 95 | 90 | 20 | 9.7 | 8.7 | 8.3 | 0.2 | 10.6 | 0.0 | 115.9 | 0.3 | 3.3 |
|  | Maltipatpur | 7.9 | 3246 | 2345 | 625 | 600 | 162 | 47.4 | 328 | 235 | 0.2 | 432.5 | 264.2 | 762.5 | 0.3 | 22.5 |
|  | Malud | 7.2 | 638 | 471 | 125 | 175 | 42 | 17.0 | 42.8 | 41.3 | 0.1 | 70.9 | 26.6 | 152.5 | 2.0 | 63.7 |
|  | RebanaNuagaon | 7.5 | 582 | 425 | 183 | 195 | 46 | 19.4 | 28.8 | 18.8 | 0.1 | 35.5 | 25.7 | 223.3 | 0.3 | 11.9 |
|  | Ramchandi | 7.3 | 562 | 365 | 85 | 200 | 50 | 18.2 | 31.3 | 5.9 | 0.2 | 95.7 | 23.2 | 103.7 | 0.2 | 23.7 |
|  | Satyabadi-1 | 8.1 | 1936 | 1441 | 375 | 350 | 58 | 49.8 | 315 | 20 | 2.9 | 322.6 | 167.9 | 457.5 | 0.8 | 8.7 |
|  | P.H.E.O.-E.E. | 8.0 | 1288 | 934 | 285 | 440 | 62 | 69.3 | 103 | 20.9 | 0.9 | 124.1 | 182.7 | 347.7 | 0.2 | 0.7 |

**Appendix-BPhysiochemical parameters in groundwater for pre-monsoon season (Raw data, Supplementary Material)**

| **District** | **Location** | **pH** | **EC** | **TDS** | **TA** | **TH** | **Ca^2+^** | **Mg^2+^** | **Na^+^** | **K^+^** | **Fe^2+^** | **Cl^-^** | **SO_4_^2-^** | **HCO_3_^-^** | **F^-^** | **NO_3_^-^** |
| --- | --- | --- | --- | --- | --- | --- | --- | --- | --- | --- | --- | --- | --- | --- | --- | --- |
| **Puri**  **(PRM)** | Balanga-2 | 7.4 | 1093 | 826 | 385 | 310 | 70 | 33 | 117 | 4 | 3 | 106 | 9 | 470 | 0 | 2 |
|  | Bentapur | 7.8 | 1391 | 898 | 250 | 300 | 100 | 12 | 94 | 96 | 0 | 206 | 19 | 305 | 1 | 50 |
|  | Jharlingi | 7.7 | 1186 | 786 | 200 | 210 | 62 | 13 | 166 | 11 | 1 | 227 | 46 | 244 | 0 | 3 |
|  | Kalyanpur | 7.0 | 17028 | 11230 | 210 | 3280 | 640 | 408 | 2400 | 35 | 1 | 5374 | 465 | 256 | 1 | 1 |
|  | Konark | 7.0 | 398 | 298 | 125 | 150 | 40 | 12 | 15 | 12 | 0 | 43 | 8 | 153 | 0 | 9 |
|  | Maltipatpur | 7.7 | 2885 | 2135 | 690 | 700 | 174 | 64 | 212 | 230 | 0 | 443 | 61 | 842 | 0 | 31 |
|  | Malud | 6.7 | 864 | 649 | 195 | 235 | 72 | 13 | 53 | 49 | 0 | 99 | 18 | 238 | 1 | 97 |
|  | Puri Town | 7.9 | 1162 | 718 | 265 | 300 | 42 | 47 | 90 | 19 | 1 | 135 | 27 | 323 | 0 | 0 |
|  | RebanaNuagaon | 8.1 | 535 | 393 | 160 | 165 | 40 | 16 | 32 | 25 | 0 | 53 | 16 | 195 | 0 | 4 |
|  | Ramchandi | 7.1 | 677 | 425 | 125 | 240 | 60 | 22 | 28 | 17 | 0 | 128 | 7 | 153 | 0 | 1 |
|  | Satyabadi-1 | 7.8 | 2748 | 1821 | 360 | 400 | 90 | 43 | 458 | 25 | 2 | 652 | 67 | 439 | 1 | 8 |

**Appendix-C Physiochemical parameters in groundwater for monsoon season (Raw data, Supplementary Material)**

| **District** | **Location** | **pH** | **EC** | **TDS** | **TA** | **TH** | **Ca^2+^** | **Mg^2+^** | **Na^+^** | **K^+^** | **Fe^2+^** | **Cl^-^** | **SO_4_^2-^** | **HCO_3_^-^** | **F^-^** | **NO_3_^-^** |
| --- | --- | --- | --- | --- | --- | --- | --- | --- | --- | --- | --- | --- | --- | --- | --- | --- |
| **Puri**  **(MON)** | Balanga-2 | 7.5 | 962 | 688 | 270 | 315 | 76 | 30.4 | 81 | 3.7 | 3.4 | 109.9 | 37.7 | 329.4 | 0 | 2.7 |
|  | Bentapur | 7.5 | 1258 | 885 | 235 | 325 | 68 | 37.7 | 88.5 | 91.8 | 0.1 | 212.7 | 20.8 | 286.7 | 0.71 | 60.3 |
|  | Gop-3 | 7.9 | 423 | 307 | 125 | 130 | 26 | 15.8 | 37.3 | 3.3 | 0.6 | 42.5 | 8.5 | 152.5 | 0.01 | 13.3 |
|  | Jharlingi | 7.7 | 384 | 292 | 150 | 150 | 52 | 4.9 | 14.2 | 6.2 | 0.8 | 21.3 | 4.3 | 183 | 0.05 | 3.4 |
|  | Kalyanpur | 7.5 | 924 | 699 | 295 | 270 | 86 | 13.4 | 80.1 | 21.4 | 0.2 | 95.7 | 16.6 | 359.9 | 0.28 | 11.4 |
|  | Konark | 7.9 | 559 | 383 | 130 | 200 | 46 | 20.7 | 22.9 | 13.8 | 0.1 | 63.8 | 10.2 | 158.6 | 0.04 | 34.8 |
|  | Maltipatpur | 8.4 | 3374 | 2461 | 650 | 730 | 174 | 71.7 | 285 | 270 | 0.2 | 436.0 | 284.2 | 780.8 | 0.06 | 86.8 |
|  | Malud | 7.3 | 834 | 621 | 145 | 260 | 84 | 12.2 | 48.8 | 47.1 | 0.4 | 102.8 | 18.9 | 176.9 | 0.71 | 123.1 |
|  | RebanaNuagaon | 7.7 | 299 | 219 | 80 | 90 | 26 | 6.1 | 13.9 | 16.9 | 1.1 | 17.7 | 18.0 | 97.6 | 0.06 | 15.0 |
|  | Ramchandi | 7.9 | 361 | 268 | 90 | 110 | 34 | 6.1 | 26.9 | 17.7 | 0.3 | 53.2 | 7.8 | 109.8 | 0.00 | 4.6 |
|  | Satyabadi-1 | 8.5 | 2648 | 1751 | 365 | 370 | 120 | 17.0 | 430 | 24 | 1.6 | 613.3 | 55.2 | 420.9 | 0.73 | 8.6 |
|  | P.H.E.O.-E.E. | 8.2 | 1173 | 814 | 330 | 345 | 96 | 25.5 | 89.3 | 20.6 | 0.8 | 131.2 | 29.9 | 402.6 | 0.00 | 1.4 |

**Appendix-D Physiochemical parameters in groundwater for post-monsoon season (Raw data, Supplementary Material)**

| **District** | **Location** | **pH** | **EC** | **TDS** | **TA** | **TH** | **Ca^2+^** | **Mg^2+^** | **Na^+^** | **K^+^** | **Fe^2+^** | **Cl^-^** | **SO_4_^2-^** | **HCO_3_^-^** | **F^-^** | **NO_3_^-^** |
| --- | --- | --- | --- | --- | --- | --- | --- | --- | --- | --- | --- | --- | --- | --- | --- | --- |
| **Puri**  **(POM)** | Balanga-2 | 7.3 | 1128 | 838 | 405 | 395 | 100 | 35.2 | 72 | 3.8 | 0.6 | 106.4 | 6.9 | 494.1 | 0 | 1.8 |
|  | Bentapur | 7.5 | 703 | 478 | 170 | 165 | 42 | 14.6 | 45.3 | 52 | 0.8 | 92.2 | 7.5 | 207.4 | 0.69 | 6.9 |
|  | Gop-3 | 8.0 | 599 | 411 | 150 | 155 | 44 | 10.9 | 58 | 10 | 0.2 | 81.5 | 2.6 | 183 | 0.19 | 3.9 |
|  | Jharlingi | 7.7 | 418 | 326 | 155 | 130 | 40 | 7.3 | 37.7 | 4.98 | 1.3 | 28.4 | 9.4 | 189.1 | 0 | 2.6 |
|  | Kalyanpur | 7.5 | 784 | 578 | 230 | 195 | 14 | 38.9 | 79.9 | 16.9 | 0.1 | 85.1 | 14.0 | 280.6 | 0.12 | 20.9 |
|  | Konark | 7.6 | 338 | 255 | 125 | 90 | 24 | 7.3 | 16.3 | 18.3 | 0.3 | 14.2 | 2.4 | 152.5 | 0.02 | 13.8 |
|  | Maltipatpur | 7.5 | 3097 | 2315 | 725 | 625 | 180 | 42.5 | 276 | 274 | 0.1 | 432.5 | 51.5 | 884.5 | 0.05 | 123.1 |
|  | Malud | 7.3 | 611 | 463 | 170 | 160 | 50 | 8.5 | 45.1 | 38.2 | 0.1 | 70.9 | 21.9 | 207.4 | 0.98 | 11.4 |
|  | RebanaNuagaon | 7.6 | 477 | 361 | 160 | 155 | 50 | 7.3 | 26.9 | 16.9 | 0.1 | 31.9 | 10.8 | 195.2 | 0 | 11.9 |
|  | Ramchandi | 7.7 | 375 | 246 | 55 | 90 | 22 | 8.5 | 29.9 | 16.9 | 0.1 | 63.8 | 8.0 | 67.1 | 0 | 21.4 |
|  | Satyabadi-1 | 8.1 | 1999 | 1432 | 375 | 310 | 62 | 37.7 | 325 | 18 | 2.1 | 343.9 | 130.9 | 457.5 | 0.76 | 8.1 |
|  | P.H.E.O.-E.E. | 7.9 | 1192 | 774 | 310 | 310 | 82 | 25.5 | 91.2 | 19.2 | 0.5 | 138.3 | 24.2 | 378.2 | 0 | 0.0 |
